# Antimalarial Activity of Piperine

**DOI:** 10.1155/2018/9486905

**Published:** 2018-12-06

**Authors:** Artitaya Thiengsusuk, Phunuch Muhamad, Wanna Chaijaroenkul, Kesara Na-Bangchang

**Affiliations:** ^1^Drug Discovery and Development Center, Thammasat University (Rangsit Campus), Pathumtani 12121, Thailand; ^2^Center of Excellence in Pharmacology and Molecular Biology of Malaria and Cholangiocarcinoma, Chulabhorn International College of Medicine, Thammasat University, Pathumthani 12121, Thailand

## Abstract

Malaria remains a public health problem in tropical and subtropical regions. Resistance of* Plasmodium falciparum* to artemisinins in Southeast Asia is a great concern for disease control and research on discovery and development of new alternative antimalarial drugs is urgently required. In a previous study, the fruit of* Piper chaba* Hunt. was demonstrated to exhibit promising antimalarial activity against the asexual stage of 3D7 (chloroquine-sensitive) and K1 (chloroquine-resistant)* P. falciparum* clones. The aim of the present study was to further investigate the antimalarial activity of piperine, the major isolated constituent of* Piper chaba* Hunt. fruits against both* P. falciparum* clones. The antimalarial activity was determined using SYBR green-I-based assay and morphological change was observed under the light microscope with Giemsa staining. The median IC_50_ (concentration that inhibits parasite growth by 50%) values of piperine against 3D7 and K1* P. falciparum *were 111.5 and 59 *μ*M, respectively. A marked change in parasite morphology was observed within 48 hours of piperine exposure. Results of real-time PCR showed no effect of piperine on modulating the expression of the three genes associated with antimalarial drug resistance in* P. falciparum*,* i.e*.,* pfcrt*,* pfmdr1*, and* pfmrp1*. Piperine could be a promising candidate for further development as an antimalarial drug based on its antimalarial potency and low risk of resistance development.

## 1. Introduction

The emergence and spread of artemisinin-resistant* Plasmodium falciparum* is a major concern for malaria control in malaria-endemic areas particularly in areas bordering Myanmar and Cambodia [1, 2]. In Thailand and most countries in Southeast Asia, a three-day artesunate-mefloquine combination has been used as first-line treatment for acute uncomplicated* P. falciparum* malaria. Nevertheless, accumulating evidence of delayed parasite clearance with reduced parasite sensitivity has been reported in Cambodia and other countries in Greater Mekong Subregion since 2003 [3–9]. In the light of these reports, research on discovery and development of effective alternative antimalarial candidates are urgently required.

Piperine is a major amide isolated from the fruits of* Piper chaba* Hunt. This compound has been demonstrated to possess several biological activities including immunomodulatory, antioxidant, antiasthmatic, anticarcinogenic, antipyretic, anti-inflammatory, antiulcer, antidepressive, and antiamoebic activities [10]. Moderate antimalarial activity of the ethanolic extract of* Piper chaba* Hunt. (fruits) has been demonstrated in our previous study [11] with IC_50_ (concentration that inhibits parasite growth by 50%) of 5.3 and 4.1 *μ*g/ml in K1 and 3D7* P. falciparum* clones, respectively. The aim of the present study was to further investigate the antimalarial activity of piperine, the major isolated constituent of* Piper chaba* Hunt. against both* P. falciparum *clones. In addition, its modulatory effects on the expression of the key genes associated with antimalarial drug resistance were also investigated. These included* Plasmodium falciparum* multidrug resistance 1 (*pfmdr1*),* Plasmodium falciparum* multidrug resistance protein 1 (*pfmrp1*), and* Plasmodium falciparum* chloroquine resistance transporter (*pfcrt*).

## 2. Materials and Methods

### 2.1. Parasites, Chemicals, and Reagents

Mefloquine, chloroquine, artesunate, and diethylpyrocarbonate (DEPC) were purchased from Sigma-Aldrich Inc. (St. Louis, MO, USA). Piperine (98%purity) was purchased from Wako Pure Chemical Co., Ltd. (Tokyo, Japan). Roswell Park Memorial Institute (RPMI) 1640, HEPES, and gentamicin were supplied by Gibco BRL Life Technologies (Grand Island, NY, USA). SYBR Green I was purchased from Sigma-Aldrich Inc. (St. Louis, MO, USA). Ethanol was purchased from Labscan Co. Ltd. (Bangkok, Thailand). All reference compounds and piperine were prepared as stock solutions of 10 mM and 3.5 mM in 50% ethanol, respectively.

### 2.2. *In Vitro* Antimalarial Activity of Piperine, Chloroquine, Mefloquine, and Artesunate

Two* P. falciparum* parasite clones,* i.e.,* 3D7 (chloroquine-sensitive) and K1 (chloroquine- resistant), were used in the study. These two parasite clones were kindly provided by the Liverpool School of Tropical Medicine, UK, and School of Public Health, Chulalongkorn University, Thailand, respectively. Both were maintained in continuous culture in O^+^ human erythrocytes suspended in RPMI culture medium supplemented with 10% human B serum and 25 mM HEPES (at 37°C under 5% CO_2_, 5% O_2_, and 90% N_2_ atmosphere) [12]. The level of parasitemia in the culture was maintained at 2-5%. Synchronization of the parasite culture to ring stage* P. falciparum *was obtained using 5% sorbitol [13].

For antimalarial activity evaluation, highly synchronous ring stage* P. falciparum* was used in each assay. An aliquot of parasite inoculum (50 *μ*l) with 2% parasitemia and 1% hematocrit was added to each well of a 96-well microtiter plate. The plate was predosed with the test compounds at eight final concentrations in the concentration ranges of 2.74-350 *μ*M (piperine), 1.63-200 nM (mefloquine), 3.9-500 nM (chloroquine), and 0.39-50 nM (artesunate). The plates were incubated at 37°C under 5% CO_2_, 5% O_2_, and 90% N_2_ atmosphere for 48 h. The IC_50_ value (concentration that inhibits cell growth by 50%) was used as an indicator of the antimalarial potency of each compound and was determined from the log-concentration-response curve analysis using Calcusyn™ software version 1.1 (BioSoft, Cambridge, UK). Data are presented as median (range) values of three independent experiments (triplicate each).

### 2.3. Morphological Change of Parasite Cells following Exposure to Piperine

Synchronized 3D7* P. falciparum* was used in the experiment. The parasite was exposed to piperine at the concentrations of IC_50_ (111.5 *μ*M) and IC_90_ (329 *μ*M) at 37°C under 5% CO_2_, 5% O_2_, and 90% N_2_ atmosphere for 48 h. Blood film slides were prepared at the following time points 2, 4, 8, 12, 24, 36, and 48 h and stained with Giemsa (Biotechnical Thai, Bangkok, Thailand). Parasite cell morphology was observed under the light microscope (x100, Olympus, Tokyo, Japan).

### 2.4. Parasite Gene Expression following Exposure to Piperine

#### 2.4.1. Preparation of RNA 

The 3D7* P. falciparum* clone was exposed to piperine as previously described in 2.3. Parasite cell pellets were separated from cell suspension following 2, 4, 8, 12, 24, 36, and 48 h of exposure. Total RNA was extracted using Trizol™ reagent according to the manufacturer's protocol (Ambion, California, USA). Briefly, the parasite was lysed with 0.5 ml of Trizol reagent in 500 *μ*l parasite suspension, thoroughly mixed and incubated at 37°C under 5% CO_2_, 5% O_2_, and 90% N_2_ atmosphere for 5 min. Two ml of chloroform was added and parasite cell suspension was incubated at 25°C for 3 min. Cell debris was removed by centrifugation (1,372 xg for 30 min, 4°C) and the supernatant was transferred to a new tube. The parasite RNA was precipitated by adding isopropanol and incubated overnight at 4°C. RNA pellets were separated through centrifugation (21,952 xg for 30 min, 4°C) and washed with 800 *μ*l of cold 75% ethanol for three times. The pellets were dried and reconstituted with 20 *μ*l of DEPC-treated water. The concentration of RNA was determined using NanoDrop Spectrophotometry (NanoDrop Technologies, Wilmington DE, USA). The contaminating DNA was removed by treatment with RQ1 RNase-Free DNase according to the manufacturer's protocol (Promega, Mannheim, Germany).

#### 2.4.2. Preparation of First-Strand cDNA Synthesis

The cDNA of each parasite clone was prepared using SuperScript® VILO™ cDNA Synthesis Kit according to the manufacturer's protocol (Invitrogen, Karlsruhe, Germany). Briefly, total reaction volume was gently mixed and incubated at 25°C for 10 min, followed by 42°C for 60 min. The reaction was terminated by heating at 85°C for 5 min. The cDNA was synthesized in a total volume of 20 *μ*l containing 5x VILO™ Reaction mix, 10x Superscript® enzyme mix, DEPC water, and parasite RNA (2 *μ*g).

#### 2.4.3. Quantification of the Expression of* pfmdr1*,* pfmrp1*, and* pfcrt* Genes

The expression of* pfmdr1*,* pfmrp1*, and* pfcrt* genes of both* P. falciparum* clones was determined by SYBR Green I real-time PCR (iCycler, Bio-Rad, USA) using the default thermocycler program: denaturation at 95°C for 5 min followed by 40 cycles of amplification at 95°C for 15 sec and annealing at 60°C for 1 min. Individual real-time PCR reaction was carried out in a 25 *μ*l reaction volume in a 96-well plate containing 10 *μ*M each of sense and antisense primer, 12.5 *μ*l of Platinum® SYBR Green qPCR SuperMix-UDG (Invitrogen, California, USA), and 50 ng cDNA. Each RT-PCR was performed in duplicate. Ct values (threshold cycle) which is the intersection between an amplification and threshold line was generated to reflect relative measure of the concentration of target in the RT-PCR reaction. The forward and reverse primers used in the experiment are shown in Table 1.

The 2^−ΔΔCt^ method of relative quantification was adapted to estimate gene expression in* P. falciparum*. The ΔΔCt method was used to calculate MDR-1, CRT, and MRP1 gene expression levels relative to control and the house-keeping gene *β*-actin was used for normalization of MDR-1 expression. The delta-delta Ct calculation for the relative quantification of the target gene was as follows:

  ΔCt (1) = [Ct (target gene) - Ct (*β*-actin)]
  ΔCt (2) = [Ct control for MDR – 1) - Ct (control for *β*-actin)]
  ΔΔCt = ΔCt (1) - ΔCt (2)
  Relative expression =2^−ΔΔCt^

Each individual sample was analyzed in triplicate and the Ct of each well was recorded at the end of the reaction.

## 3. Results

### 3.1. Antimalarial Activities of Piperine, Chloroquine, Mefloquine, and Artesunate

**Figure 1:**
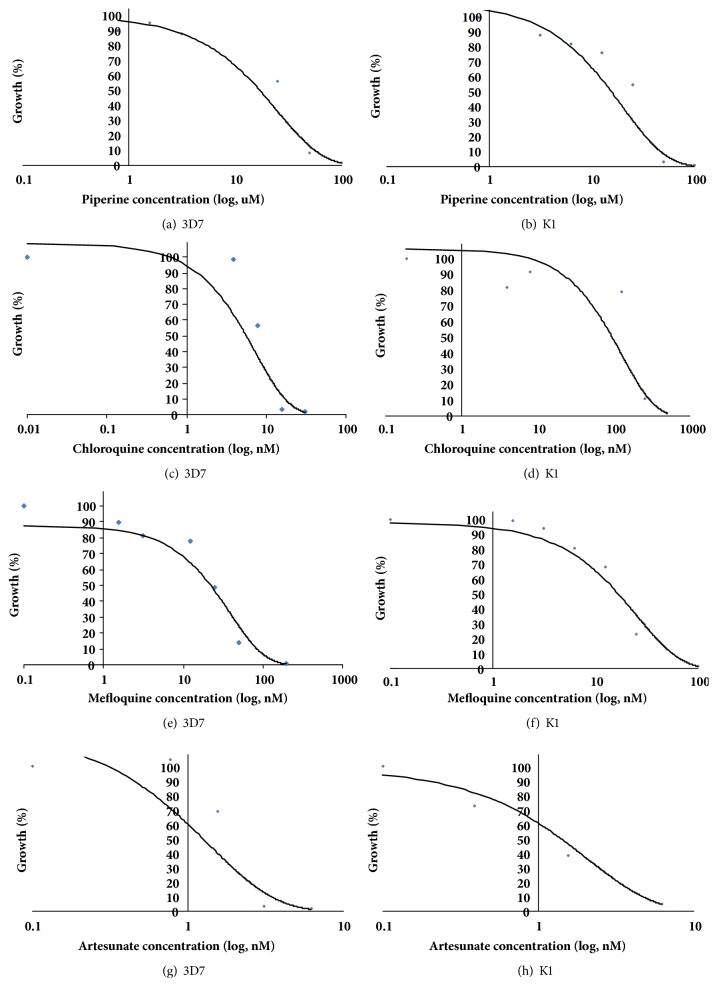
Concentration-response curves of 3D7 and K1* P. falciparum* clones following exposure to piperine (a, b), chloroquine (c, d), mefloquine (e, f), and artesunate (g, h). Data are presented as the median value of three independent experiments, triplicate each.

The median IC_50_ (range) values of piperine, chloroquine, mefloquine, and artesunate for 3D7 and K1* P. falciparum* are summarized in Table 2 and the concentration-response curves of each compound for both* P. falciparum* clones are shown in Figures 1(a)–1(h).

### 3.2. Morphological Change of Parasite following Exposure to Piperine

**Figure 2:**
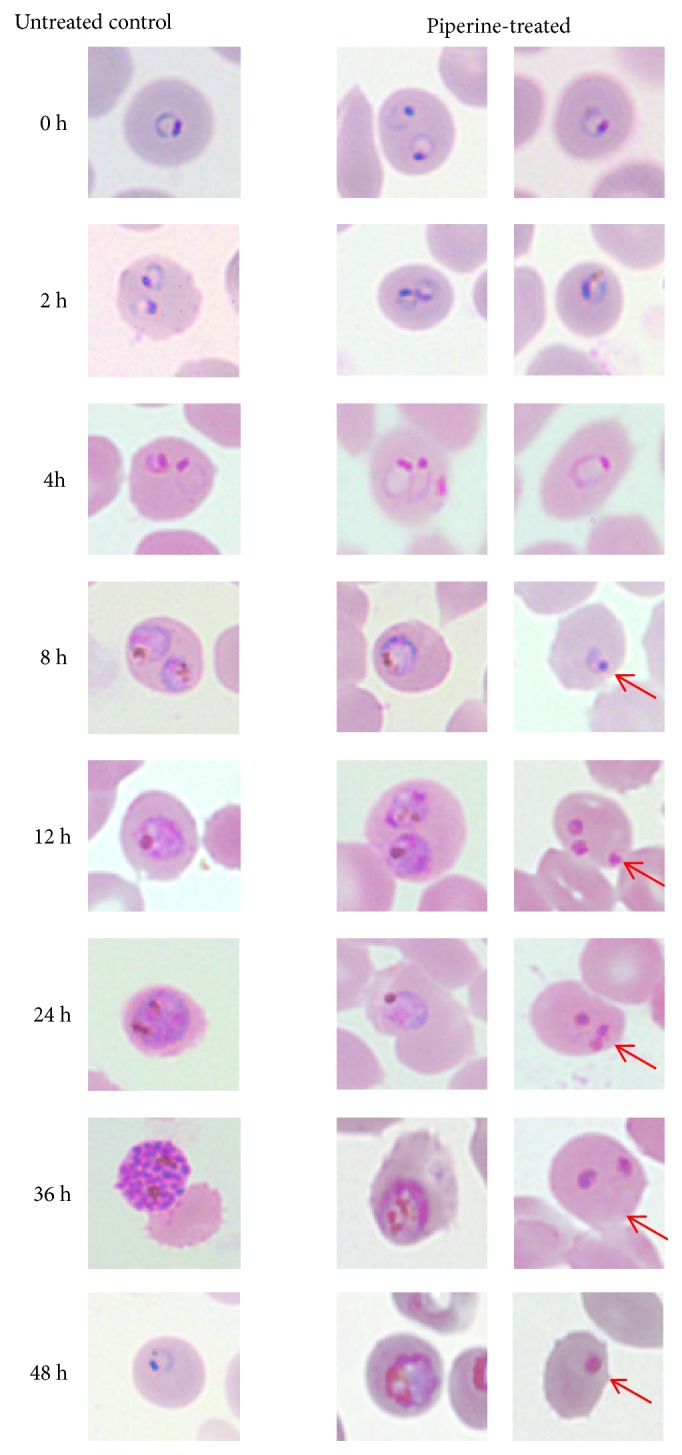
Giemsa-stained thin blood films of 3D7* P. falciparum* after exposing to piperine (at IC_50_) in comparison with untreated control parasite during 0-48 hours period. The red arrow indicates the change in morphology of the exposed parasites.

The time- and stage-specific antimalarial actions of piperine were investigated using 3D7* P. falciparum* clone. The morphological changes of 3D7* P. falciparum* following exposure to piperine at 111.5 *μ*M (IC_50_) and 329 *μ*M (IC_90_) were observed compared with untreated control parasite during the period of 0 to 48 hours. The change started from 8 hours but the effect was clearly seen at 12 hours of piperine exposure (Figure 2). The parasite growth was slowed down and the cytoplasm was condensed compared with untreated cells. In addition, some of the surviving parasites showed a slower growth rate. At IC_90_, almost all parasites died after 8 hours of piperine exposure.

### 3.3. Expression of* pfmdr1*,* pfmrp1*, and* pfcrt* Genes following Exposure to Piperine

The expression of* pfmdr1*,* pfmrp1*, and* pfcrt* of 3D7* P. falciparum* following exposure to piperine at the IC_50_ (111.5 *μ*M) and IC_90_ (329 *μ*M) was observed at 2, 4, 8, 12, 24, 36, and 48 hours. No change in the expression of all genes was found in both piperine-treated and untreated control parasites at each exposure time point.

## 4. Discussion

The IC_50_ of the crude ethanolic extract of* Piper chaba* Hunt. (fruits) reported in our previous study in K1 and 3D7* P. falciparum* clones were 5.3 and 4.1 *μ*g/ml, respectively [11]. The antimalarial potencies of piperine against both chloroquine-sensitive (3D7) and chloroquine-resistant (K1)* P. falciparum *clones are considered low compared with the standard antimalarial drugs under investigation. Results of the morphological study suggest that the window of activity of piperine is likely to be the late ring to trophozoite stages (8-12 h). Further investigation to elucidate the mechanism of action of piperine at molecular and cellular levels is underway. It was noted however that the potencies of activity of the isolated compound piperine (IC_50_: 59 and 111.5 *μ*M for K1 and 3D7, respectively) were relatively low compared with the crude ethanolic extract in both clones. This suggests that antimalarial activity of the extract is a result of additive or synergistic interaction of various constituents in the plant extract. The reported isolated compounds from* Piper chaba* Hunt. include piperine, N-isobutyl amide of octadeca-trans-2-cis-4-dienoic acid [14], isopiperolein B [15], piperchabamide D, dehydropipermolanine, and dehydropopermoline. Moreover, pipernonaline, guineensine, isobutylamide of 11-(3,4-methylenedioxy-phenyl)undeca-2,4,10-trienoic acid, piperchabamides A, piperchabamides B, piperchabamides C, piperlonguminine, retrofractamide B, N-isobutyl-(2E,4E,14Z)-eicosatrienamide, and methylpiperate were also identified [16]. The* in vivo *antimalarial activity of piperine, when given in combination with curcumin, was reported in* Plasmodium chabaudi-*infected mouse model. Piperine (oral dosing of 20 mg/kg body weight/day for 15 days) used as an enhancer for improving the bioavailability of curcumin (oral dosing of 300 mg/kg body weight/day for 15 days) showed moderate antimalarial activity. Combining piperine (20 mg/kg body weight/day) with curcumin (300 mg/kg body weight/day) and artemisinin (150 mg/kg body weight/day) for 15 days provided no additional benefit on efficacy improvement [17]. In another* in vivo* study in AS-3CQ (chloroquine-resistant) strain of* P. chabaudi*, the combination of piperine (20 mg) with curcumin (250 mg) and chloroquine (2.5 mg) was given for 4 days and followed up for 7 days and was shown to produce additive effect in reducing the parasite load 7 days after treatment. The combination was effective in reducing parasitemia to 45% in mice infected with chloroquine-resistant AS-3CQ* P. chabaudi* and to 44% in mice infected with artemisinin-resistant AS-ART* P. chabaudi* [18]. Altogether, these results suggest low to moderate antimalarial activity of piperine both* in vitro* and in animal models.

*Pfmrp1*,* pfmdr1*, and* pfcrt* genes of* P. falciparum* have been reported to be associated with the decrease in clinical efficacy of several antimalarial drugs. Compounds or drugs which modulate the expression of these genes may pose the risk of resistance development in the treatment of* P. falciparum*. The* P. falciparum* multidrug resistance protein 1 (*pfmrp1*) gene that encodes the PfMRP1 protein is a member of the ABC transporter superfamily located on chromosome 1 [19]. Increased expression of* pfmrp1 *gene has been associated with mefloquine, quinine, and chloroquine resistance. Moreover, this protein has been reported to be involved in the export of folate from malaria parasites into red blood cells [20]. The* P. falciparum* multidrug resistance gene 1 (*pfmdr1*) that encodes a P-glycoprotein homologue is also located on the membrane of parasite digestive vacuole, the main target of action of most antimalarial drugs. It is thought to play a role in modulation of the level of antimalarial drug resistance [21] by transporting drugs from parasite cytosol to digestive vacuole. The introduction of gene polymorphisms results in chloroquine and quinine-resistant phenotypes [22]. Finally, the* P. falciparum* chloroquine resistance transporter (*pfcrt*) encodes a protein localized on the membrane of parasite digestive vacuole and contains 10 predicted membrane-spanning domains in the erythrocytic stage of* P. falciparum *[23]. The K76T mutation has been linked to chloroquine resistance in parasite isolates collected worldwide [24]. Based on the results of the present study, no effects of piperine were found on modulating (inducing or inhibiting) the expression of all* P. falciparum* resistance genes under investigation including* pfmrp1*,* pfmdr1*, and* pfcrt*. This may imply a low risk of resistance development of* P. falciparum* by piperine.

## 5. Conclusions

Piperine could be a promising candidate for further development as an antimalarial drug with regard to its antimalarial potency and low tendency to modulate* P. falciparum* resistance genes and thus resistance development.

## Figures and Tables

**Table 1:** The forward and reverse primers used for quantification of *pfmdr, pfmrp1,* and *pfcrt* gene expression.

Gene	Sequence (5'-3')
*pfmdr1*	Forward CAAGTGAGTTCAGGAATTGGTAC
	Reverse ATG GCCTCTTCTATAATGGACATGG
*pfmrp1*	Forward AGTAGAAGGAAGAGACATTCGAACATA
	Reverse CAAAAGAAGATTGAGCTAAAATACCAA
*Pfcrt*	Forward CCCAAGAATAAACATGCGAAAC
	Reverse ACAATTATCTCGGAGCAGTT
*Pfβ-actin*	Forward CCAGCTATGTATGTTGCTATTC
	Reverse CTCCACTATCTAACACAATACC

**Table 2:** Antimalarial activities expressed as IC_50_ values of piperine, chloroquine, mefloquine, and artesunate in 3D7 and K1 *P. falciparum* clones. Data are presented as median (range) value of three independent experiments, triplicate each.

Compounds	Median IC_50_ (range)	
	3D7	K1
Piperine (uM)	111.5 (103.3-117.0)	59.0 (55.0-70.0)
Chloroquine (nM)	3.9 (3.5-4.0)	115.0 (114.9-135.4)
Mefloquine (nM)	14.6 (13.8-17.3)	4.22 (3.93-4.83)
Artesunate (nM)	2.48 (1.84-3.01)	1.06 (1.06-1.17)

## Data Availability

The data used to support the findings of this study are available from the corresponding author upon request.
